# Evaluation of the Workplace-Based Assessment Anaesthesia-Clinical Evaluation Exercise (A-CEX) and Its Role in the Royal College of Anaesthetists 2021 Curriculum

**DOI:** 10.7759/cureus.37402

**Published:** 2023-04-10

**Authors:** Harry Bamber

**Affiliations:** 1 Anaesthesia, Glan Clwyd Hospital, Rhyl, GBR

**Keywords:** meded, wbpa, anaesthesia, cex, education, medical

## Abstract

The workplace-based assessment (WPBA) Anaesthesia-Clinical Evaluation Exercise (A-CEX) is used in anaesthetic training in the Royal College of Anaesthetists 2021 curriculum. WBPAs are part of a multimodal approach to assess competencies, but can be limited by their granularity. They are an essential component of assessment and are used in both a formative and summative capacity. The A-CEX is a form of WBPA which evaluates knowledge, behaviours and skill of anaesthetists in training across a variety of ‘real world’ situations. An entrustment scale is assigned to the evaluation which has implications for future practice and ongoing supervision requirements. Despite being a key component in the curriculum the A-CEX has drawbacks. Its qualitative nature results in variation in feedback provided amongst assessors, which may have ongoing implications for clinical practice. Furthermore, the completion of an A-CEX can be viewed as a ‘tick box' exercise and does not guarantee that learning has taken place.

Currently no direct evidence exists as to the benefit of the A-CEX in anaesthetic training, but extrapolated data from other studies may show validity. However, the assessment remains a key part of the 2021 curriculum,

Future areas for consideration include education for those assessing trainees via A-CEX, altering the matrix of assessment to a less granular approach and a longitudinal study as to the utility of A-CEX in anaesthetics training.

## Introduction and background

Context

Anaesthetic training in the United Kingdom is overseen by the Royal College of Anaesthetists (RCoA). In 2021, an updated curriculum was released for trainees to obtain a certificate of completion in training (CCT) [1]. Key stakeholders of this curriculum include the regulatory body for doctors, the General Medical Council (GMC), the independent regulatory body, and the Professional Standards Authority (PSA) as well as current anaesthetic trainees and consultant anaesthetists. 

Workplace-based assessments (WPBA) are a core feature of this curriculum and therefore anaesthetics training, both in a formative and summative capacity.

Overview of the 2021 RCoA curriculum including rationale of WPBA

The purpose of the anaesthetic curriculum is to develop doctors with generic and specialty-specific capabilities to lead, develop and deliver high-quality anaesthesia [1]. The curriculum is outcome based using 14 domains delivered over three stages. It uses two broad assessment tools: RCoA examinations and WPBA. 

**Figure 1:**
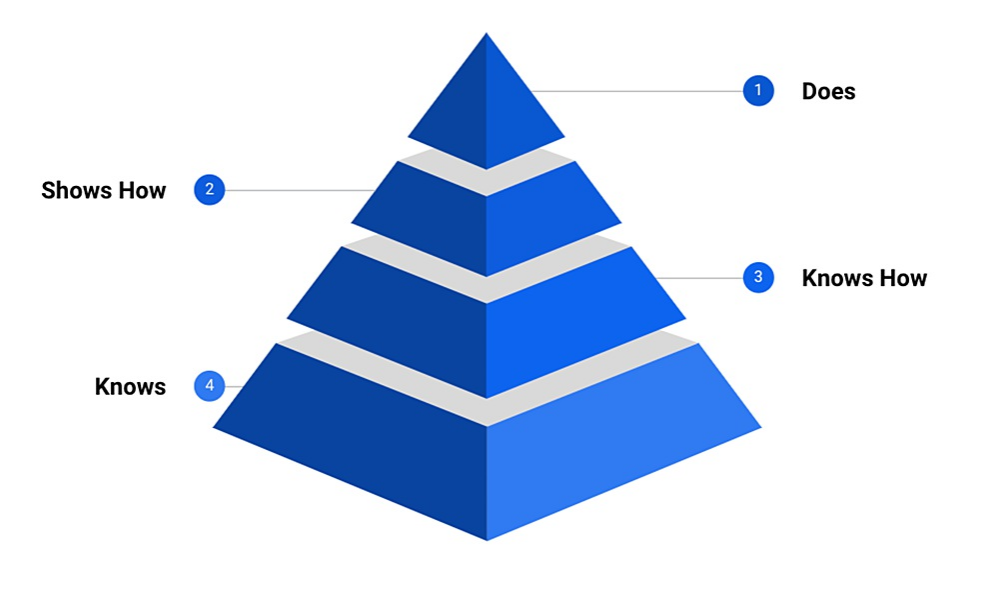
Miller's pyramid [2]

There are several different examination formats that contribute to critical progression points in the curriculum, each specifically designed to assess knowledge with respect to Miller’s pyramid (Figure 1). True/False and single best answer questions assess ‘Knows’ and ‘Knows how’, whilst the objective structured clinical examinations (OSCE) assess ‘shows how’.

WPBAs on the other hand are used for observed clinical practice and therefore represent ‘Does’. They are both formative and summative. This is vitally important in both trainee development and assessment as it bridges the gap between theoretical knowledge which may be learnt solely for examinations and ‘real word’ clinical practice underpinned by knowledge, technical skills and non-technical skills. In other words, it looks to measure a trainee’s performance in a clinical environment. Rethans et al. propose that clinical performance is a function of both systemic and individual influences. Systemic influences include staffing levels, governance processes and the availability of resources such as investigation results and guidelines. Individual influences might be current level of fatigue, hunger status and health [3]. Currently, services are increasingly stretched and this has subsequent effects on performance. Each assessment, therefore, requires a nuanced approach with an appreciation of clinical context. However, this jeopardises standardisation and makes comparison between trainees or even departments challenging which can have governance implications. Ultimately, since the rationale behind WPBAs is ‘Does’, the wider context should be included. This is addressed, in part, by the Entrustable Professional Activities (EPA) model discussed shortly.

Influence on learners and stakeholders

There are several stakeholders involved in anaesthetic training and the anaesthetic curriculum design. These include trainees/trainers (via curriculum surveys, trainee groups and education fellowship posts), the GMC/PSA, the Department of Health and service users. In his review of the 2010 anaesthetic curriculum, Dr Aiden Devlin suggested that there was an excessive reliance on WPBAs, leading to a ‘tick-box culture’, whereby trainees would obtain WPBAs for the sake of meeting requirements, rather than as a true assessment of learning [4]. Another finding was the unofficial summative nature of the WPBAs as trainees could be graded ‘satisfactory’ or ‘unsatisfactory’. This led to trainees only approaching trainers once they were certain of a satisfactory outcome. The 2021 Curriculum addresses these points and introduces an entrustment scale that quantifies the level of supervision required if trainees were in an identical scenario in the future. The scale is a formative tool used to demonstrate trainee progress across a timeframe. It can also be used towards summative progression points.

Although commonly used in medical education, WPBAs have limitations. They can answer specific questions, but this may lead to them becoming too narrow and granular, and the holistic clinician underneath is lost. The 2021 curriculum design attempts to mitigate this with multiple assessors, a mixture of assessment methods and moderators, to broadly assess trainees. The EPA model is the result of this data-gathering exercise and is a holistic tool that assesses trainees across different skills and behaviours, to determine suitability for unsupervised practice of designated activities. It’s a summative assessment by training faculty members, and uses WPBA as well as individual judgement regarding a trainee’s functioning against a set rubric. This helps mitigate some of the limitations of competency-based training where false positives or negatives can occur. More importantly, it provides clear descriptors of expected functioning for doctors who will have had different undergraduate training and experience to date and thus is a means of upholding standards. In the 2021 Anaesthetics Curriculum, it is used as a summative assessment to determine novice anaesthetists' competencies and abilities in real-world clinical scenarios. EPA is a relatively new addition to curricula: a 2019 review of 80 articles concluded a lack of high level evidence demonstrating its efficacy or validity in the healthcare sector [5]. However it displays theoretically desirable characteristics, and therefore seems a reasonable model to continue with pending more specific data on outcomes in anaesthetics training.

## Review

The assessment

The Anaesthesia-Clinical Evaluation Exercise (A-CEX) is a form of WPBA used in anaesthetic training in the 2021 curriculum. It is a formative assessment used during clinical practice, based on the observed practice of a trainee and evaluates knowledge, behaviours and skills.

It is completed via an electronic form which is populated by the trainee and summarises the case, including reflective discussion between trainee and trainer. The ‘ticket’ generated can be mapped to the relevant section in the curriculum via the RCoA portfolio “Lifelong Learning Platform” (LLP), thus representing constructive alignment [6].

The trainer then completes feedback with suggestions for future development and assigns an entrustment scale to the A-CEX.

Evaluation of A-CEX

The A-CEX assessment represents ‘Does’ on Miller’s pyramid (Figure 1) and captures and comments upon an anaesthetist’s actions in daily practice on a case/activity relevant to the curriculum. It is broader and more holistic than other WPBAs. For example, when comparing to a Directly Observed Practical Skills assessment (DOPS), it would ask ‘which level of supervision is required for this trainee to manage a patient in renal failure?’ versus ‘which level of supervision is required for this trainee to insert a dialysis line’. The DOPS is a more granular assessment and may not take into account broader factors that doctors encounter day-to-day such as state of mind, existing workload and finite resources/time.

The entrustment scale allows the individual trainee and trainer to see progression over time. It can also be used to compare the progression of trainees in the same hospital or across a deanery. This is important as it allows key stakeholders such as RCoA faculty to monitor local provision of anaesthetic education and to feedforward for subsequent cohorts. Most importantly though, it serves as a barometer of performance applicable to the real clinical world and thus allows provision of safe supervision for trainees. Completing A-CEXs encourages clinicians to adopt a mindset of lifelong learning where formative feedback is constantly incorporated into clinical practice for improvement and can quickly formalise discussions or learning events between trainee and trainer. In the broader sense, A-CEXs form a small piece in a mosaic of metrics to assess trainee performance and, alongside other WPBAs, contribute to summative assessments such as EPA or critical progression points in the curriculum such as the Initial Assessment of Competence (IAC), the first milestone in anaesthetic training.

Although the A-CEX has advantages, it does also present some challenges as an assessment tool. Although there is no minimum number of A-CEXs required, trainees may feel bound to complete them to satisfy a tick box process or may only submit them once they are certain they have met a minimum standard on the supervision scale [3]. As such, completion of an A-CEX does not necessarily mean that learning has taken place, especially if the feedback given is of little educational value. The system relies on the trainer and trainee having constructive alignment between objectives, educational opportunities and assessment methods. Anecdotally, in anaesthetics this has proven to be more challenging since the introduction of the new curriculum as both trainee and trainer may be unfamiliar with expected learning outcomes. This opinion has been taken from trainee meetings but lacks the formal evidence of a systematic review given the current curriculum is less than a year old.

Aside from the final entrustment rating, the feedback for an A-CEX is qualitative. This is a deliberate move by the RCoA to move away from pass/fail quantitative assessment methodology to a consensus opinion from a body of trainers. In real terms, however, this can lead to a lack of standardisation between assessments. This is especially true in anaesthesia where there is significant variation in trainers’ individual clinical practice, perhaps more so than other professions. Heterogeneity of assessment style, unconscious bias and lack of standardisation in training of trainers, may result in differing assessments of a theoretically identical performance. This can be frustrating for trainees, but can be in part mitigated against by increasing the sample breadth, both in terms of numbers and modality of WPBAs.

The utility of A-CEX

The van der Vleuten equation (Utility = Validity x Reproducibility x Equivalence x Educational and Catalytic effect x Acceptability x Fairness) [7] seeks to elucidate the components contributing to the utility of an assessment (Table 1). The components are also supported by the Ottawa consensus statement [8] and “Assessment design for learner responsibility” [9].

**Table 1:** The A-CEX with respect to the van der Vleuten equation [7] A-CEX: Anaesthesia-Clinical Evaluation Exercise, RCoA: Royal College of Anaesthetists

Component	Utility
Validity	A-CEX measures observed practice in the workplace and therefore represents ‘does’ on Miller’s pyramid. It is representative of real world clinical medicine [10]. The RCoA has ensured constructive alignment as trainees can map domains to the 2021 Curriculum.
Reproducibility	Differences between trainer assessment styles and lack of standardised training of trainers in anaesthetics leads to different A-CEX results for a given performance. Across medical education in general, there is generally poor evidence determining whether CEX provides consistency amongst different learner types across the wider clinical landscape.
Feasibility	The A-CEX is a straightforward assessment in the author’s experience and can easily be completed in the operating theatre during a case if required. However, the A-CEX relies on written feedback by a trainer. This task can fall down the list of tasks and priorities senior clinicians hold, leading to rushed or even lost feedback opportunities.
Educational effect/ Acceptability	In a meta-analysis summarising the evidence on educational impact of CEX [11] found that CEX had a positive educational impact on acquisition of knowledge and skills (Kirkpatrick level 2 [see Table 2]), but that there was no available evidence on higher Kirkpatrick levels. Studies comprising the meta-analysis compared mandatory completion of CEX vs non- mandatory or no completion on summative performance in undergraduate objective structured clinical examination (OSCE). However, this is a different population to postgraduate doctors practising clinical medicine and is a limitation to this study, albeit in a reliable and valid examination with regard to clinical competence [12]. Perceived usefulness by foundation doctors was poor (Kirkpatrick level 1), but improved with CEX-specific training [13] and CEX can help predict junior doctors in difficulty [14].

As referenced within Table 1, the Kirkpatrick Model describes the effectiveness of an educational resource (Table 2).

**Table 2:** The Kirkpatrick Model related to CEX, reproduced from Lörwald et al., 2018 [10] CEX: Clinical Evaluation Exercise

Kirkpatrick Level	Description
Level 1: Reaction	Is it favourable and relevant?
Level 2: Learning	Does it help with acquisition of knowledge and skills?
Level 3: Behaviour	To what extent does the behaviour learnt translate into a change of behaviour?
Level 4: Results	To what extent does it benefit patients?

## Conclusions

The evidence base for higher-level Kirkpatrick outcomes is, as yet, lacking for A-CEXs. Indeed some may argue that we are imposing an undesirable and unproven assessment on an overworked population. However A-CEX has some utility when studied using the van der Vleuten equation.

There is unavoidable (and necessary) heterogeneity in trainers and their opinions. However, no training is required for trainers themselves such as ‘training the trainer’ in order to complete WPBAs, nor any requirement for trainers to be education faculty members such as RCoA college tutors. It may be that stakeholder or education faculties develop EPA for themselves to help regulate and standardise this. It is difficult to determine if this would make any meaningful difference to outcomes, or indeed what those outcomes would be, but it sounds plausible that it might. This, alongside a longitudinal study into the utility of A-CEX in anaesthetics training, would be valuable.
